# A transcriptomic scan for potential candidate genes involved in osmoregulation in an obligate freshwater palaemonid prawn (*Macrobrachium australiense*)

**DOI:** 10.7717/peerj.2520

**Published:** 2016-10-05

**Authors:** Azam Moshtaghi, Md. Lifat Rahi, Viet Tuan Nguyen, Peter B. Mather, David A. Hurwood

**Affiliations:** 1 Science and Engineering Faculty, Queensland University of Technology, Brisbane, Queensland, Australia; 2 School of Science and Engineering, University of the Sunshine Coast, Sippy Downs, Queensland, Australia

**Keywords:** Transcriptome, Osmoregulation, *Macrobrachium australiense*, Hepatopancreas, Illumina, Antennal gland, Gill

## Abstract

**Background:**

Understanding the genomic basis of osmoregulation (candidate genes and/or molecular mechanisms controlling the phenotype) addresses one of the fundamental questions in evolutionary ecology. Species distributions and adaptive radiations are thought to be controlled by environmental salinity levels, and efficient osmoregulatory (ionic balance) ability is the main mechanism to overcome the problems related to environmental salinity gradients.

**Methods:**

To better understand how osmoregulatory performance in freshwater (FW) crustaceans allow individuals to acclimate and adapt to raised salinity conditions, here we (i), reviewed the literature on genes that have been identified to be associated with osmoregulation in FW crustaceans, and (ii), performed a transcriptomic analysis using cDNA libraries developed from mRNA isolated from three important osmoregulatory tissues (gill, antennal gland, hepatopancreas) and total mRNA from post larvae taken from the freshwater prawn, *Macrobrachium australiense* using Illumina deep sequencing technology. This species was targeted because it can complete its life cycle totally in freshwater but, like many *Macrobrachium* sp., can also tolerate brackish water conditions and hence should have genes associated with tolerance of both FW and saline conditions.

**Results:**

We obtained between 55.4 and 65.2 million Illumina read pairs from four cDNA libraries. Overall, paired end sequences assembled into a total of 125,196 non-redundant contigs (≥200 bp) with an N50 length of 2,282 bp and an average contig length of 968 bp. Transcriptomic analysis of *M. australiense* identified 32 different gene families that were potentially involved with osmoregulatory capacity. A total of 32,597 transcripts were specified with gene ontology (GO) terms identified on the basis of GO categories. Abundance estimation of expressed genes based on TPM (transcript per million) ≥20 showed 1625 transcripts commonly expressed in all four libraries. Among the top 10 genes expressed in four tissue libraries associated with osmoregulation, arginine kinase and Na+/K+- ATPase showed the highest transcript copy number with 7098 and 660, respectively in gill which is considered to be the most important organ involved in osmoregulation.

**Discussion:**

The current study provides the first broad transcriptome from *M. australiense* using next generation sequencing and identifies potential candidate genes involved in salinity tolerance and osmoregulation that can provide a foundation for investigating osmoregulatory capacity in a wide variety of freshwater crustaceans.

## Introduction

Currently, billions of humans rely on aquatic species as their primary source of animal protein, and so future predicted changes to fishery production are likely to have major impacts on human populations and economies worldwide (Ficke, Myrick & Hansen, 2007). Among farmed aquatic species, decapod crustacean taxa (Order: Decapoda) comprise more than 200 species (including prawns/shrimps, lobsters and crabs), many of which support significant commercial industries; of these, prawns are particularly important to the freshwater (FW) aquaculture industry. The FW prawn aquaculture industry worldwide depends largely on unimproved, essentially wild stocks for farming, most of which are produced in basic hatcheries or that are harvested as juveniles from the wild (Jung et al., 2011). Currently, the most important FW prawns used in aquaculture are all members of the genus *Macrobrachium* (Family *Palaemonidae*). *Macrobrachium* spp. display a wide variety of life history strategies and occur in a diverse array of aquatic habitats and naturally occupy a wide range of environments (including salinities) and show a variety of behavioural patterns that allow them to deal with changing environments (Huong et al., 2010). Culture of FW prawns in some places (the Mekong River Basin (MRB) is a notable example) is confronted with a serious challenge; saltwater penetration in freshwater ecosystems as many species are unable to perform osmoregulation successfully in variable environmental conditions (Nguyen et al., 2014).

FW crustaceans need to control their body ion concentrations in the face of variation in environmental salinity levels and therefore require an ability to regulate their osmotic haemolymph pressure in order to survive. The osmoregulatory system plays a pivotal role in dealing with changes in ionic concentrations (regulation of Na^+^, K^+^, H^+^, Ca^2+^, Mg^2+^, HCO${}_{3}^{-}$ and Cl^−^ ions) in an individual’s body compared with the aquatic medium they occupy. This complex interaction impacts regulation of total osmotic concentration, regulation of intracellular vs. extracellular ion levels, acid–base balance and levels of a number of other organic ions (Lignot, Spanings-Pierrot & Charmantier, 2000). This is further complicated by the presence of an open blood system.

Crustaceans are a well-known group for their successful colonisation of freshwater from the marine environment and this evolutionary trend is generally associated with a wide diversity in osmoregulatory capacity (Freire, Onken & Mcnamara, 2008). Many decapods can utilise a wide range of salinity levels (freshwater, brackish water and marine conditions), with some species able to move among ecotypes within their own life times (Gillanders et al., 2003). Therefore, efficient osmoregulatory capacity is a fundamental requirement for coping with environmental stressors and it will be important to identify physiologically significant candidate genes (CG) that contribute to effective osmoregulation in these organisms in the face of rapid environmental change. Understanding the genomic and/or functional genomic resources associated with effective salinity tolerance in crustaceans is therefore of great importance. Identifying CG loci and functional mutations within these genes that affect salinity tolerance as well as understanding how they are regulated (gene expression patterns) can assist predictive power for likely outcomes of the effects of climate change on wild populations and how impacts can be mitigated in farmed stocks.

Modern genomic approaches, particularly, next generation sequencing technology (NGST) in addition to differential gene expression (DGE) profiling allow deep understanding of gene expression patterns and signalling pathways to be developed. Transcriptomic analysis has been used widely in biological studies and has provided practical insights into gene discovery, differential gene expression patterns under different environmental conditions and also efficient discovery of molecular markers (Jin et al., 2013; Li et al., 2015; Manfrin et al., 2015; Song et al., 2015; Thanh et al., 2014; Zhao et al., 2015).

Understanding the genomic basis of salinity tolerance and relative efficiency of the osmoregulatory system under changing conditions can help us to understand the genomic basis of osmoregulation itself (Freire, Onken & Mcnamara, 2008). Salinity (ionic content) is one of the most important abiotic factors that affects species distribution, population expansions and growth of local culture industries. For instance, rates of protein synthesis and respiratory metabolism in *M. rosenbergii*, the species supporting the largest FW crustacean culture industry worldwide, are affected significantly by changes in salinity. *M. rosenbergii* individuals are very sensitive to changes in salinity, pH and temperature during the pre-moult stages of their life cycle and this can have significant effects on their immune response later (Cheng & Chen, 2000). At raised salinity levels, mortality rate is considerable and growth performance is compromised. Alternatively, in the marine shrimp *Penaeus monodon*, raised or lower levels of salinity (compared to the optimal level ∼25 ppt) is associated with increased energy expenditure for active ion transport and involves degradation of energy-rich compounds including lipids (Ye et al., 2009). These results in energy being diverted from growth to ion balance maintenance, an outcome that affects production cycle productivity.

Transcriptomic analysis can allow potential candidate genes involved in adaptation to variable osmotic environments to be identified and can determine consistent genomic changes that occur in related species across micro/macroevolutionary time scales (Jung et al., 2011). To this end here we used *Macrobrachium australiense* (Palaemonidae) as a model (to better understand potential genes involved in osmoregulation) for other freshwater prawns in the genus *Macrobrachium* because it is the most widespread FW prawns species across mainland Australia and is the most successfully adapted species to a complete freshwater life cycle (Dimmock, Williamson & Mather, 2004) yet under controlled conditions in the laboratory can tolerate up to 17 ppt salinity (Sharma & Hughes, 2009). The most important features of this model species include high tolerance of saline conditions at all life history stages, short generation time (life cycle is abbreviated into three zoeal stages, maturation normally takes 4–6 months after hatching), reduced larval stages, mature stage at hatching and relatively large egg size (Dimmock, Williamson & Mather, 2004).

Adaptation to different salinity levels is a complex process and is related to cell volume regulation, organic ion transport, changes to cellular carbohydrate levels, nitrogen metabolism and whole tissue remodelling (Henry et al., 2011). In crustaceans, in particular *Macrobrachium* prawns, certain organs/tissues provide ideal targets for a comparative genomic analysis of the molecular basis of adaptive physiological response to saline environments. Therefore, the primary aim of this study was to develop a more complete understanding of the molecular basis of osmoregulatory functioning by sequencing transcriptomes from the most important organs involved in ion regulation and salinity tolerance using an Illumina platform. The transcriptomics approach was used to identify the major potential genes involved with osmoregulation in osmoregulatory organs including the gill, antennal gland and hepatopancreas of *M. australiense*.

## Materials and Methods

### Sample collection and maintenance

*M. australiense* specimens were collected from a site on Bulimba Creek (27°56S, 153°09E) (a tributary of the Brisbane River, SE Queensland, Australia) (Field permit for this study was issued by the Department of Agriculture, Fisheries and Forestry under Queensland Government, Permit Number: 166312). This site had 0 ppt salinity but was only 22 km from the confluence with the main channel of the Brisbane River where water salinity is approximately 30 ppt. Furthermore, tidal influence occurs up to only 5 km downstream from the sampling site with no barriers to dispersal between the site and brackish water. Population genetic data (Rogl, 2015) using a COI marker revealed no evidence for genetic differentiation between different *M. australiense* populations that are separated by brackish water across the entire lower reaches of the Brisbane River drainage. It was assumed, therefore, that *M. australiense* individuals collected in this study had ready access to brackish water. Total genomic DNA was extracted from pleopods taken from each prawn for taxonomic validation using a fragment of the mtDNA COI gene following methods outlined in a previous study on *M. rosenbergii* (Hurwood et al., 2014). Sanger sequencing results for mtDNA COI confirmed that all individuals used to generate the transcriptomes in this study were *M. australiense*, with all individuals having haplotypes identical to, or minimally divergent from (one or two bp), those previously detected for this species in the Brisbane River (Sharma & Hughes, 2009).

### RNA extraction, cDNA library preparation and Illumina sequencing

For transcriptomic analyses, collected prawns were transported live to the Molecular Genetics Research Facility (MGRF) at Queensland University of Technology (QUT). Live individuals were then euthanized and dissected immediately to obtain fresh tissue for total RNA extraction. In total, 12 adult (body weight between 6–8 g) prawn individuals were used for RNA extraction but due to the small amount of available tissue, we pooled tissues from four prawns each time. Then, we used the best quality RNA samples for each tissue. In addition, we extracted total RNA from 2 post larvae (body weight 0.5–0.6 g) individuals. Dissected and pooled tissues including gill (G), antennal gland (A), and hepatopancreas (H) from adults and whole individual of post larvae (PL) were transferred to mortars containing liquid nitrogen, and tissues were crushed into a fine powder using pestles. Powdered tissues were then used for total RNA extraction using an RNeasy Mini Kit (Qiagen, Hilden, Germany) according to the manufacturer’s protocol. Genomic DNA was removed using a Turbo DNA-free kit (Ambion, Life Technologies) and the integrity and concentration of extracted total RNA was checked using a Bioanalyzer 2100 (version 6; Agilent Technologies) and a Nano Drop 2000 spectrophotometer (Thermo Scientific), respectively. RNA samples were stored at −80 °C for later use. 4 µg of total RNA were used as starting material for mRNA purification, using a TrueSeqV1 Standard mRNA Sample Prep kit (Illumina, San Diego, CA, USA). Purified mRNA was then fragmented into smaller fragments to initiate cDNA synthesis. After synthesizing the first and second strands of cDNA, an Illumina barcoding adapter (individual index) ligation step was performed chemically according to the manufacturer’s protocol. Adapter ligated cDNA was purified using the AMPure XP magnetic bead (Beckman Coulter, Danvers, MA, USA) system combined with ten subsequent washing steps. Quality of constructed cDNA libraries was checked using a Bioanalyzer 2100 (Agilent), Qubit^®^ 2.0 Fluorimeter (Invitrogen, Life Technologies) and RT-qPCR (BJS Biotechnologies, UK). All libraries were normalized via dilution before the final sequence run. The resulting four cDNA libraries (G, A, H and whole PL) were subjected to the Illumina NextSeq™ 500 Platform for 75 bp paired end sequencing. All activities were performed at the Molecular Genetics Research Facility (MGRF) under the Central Analytical Research Facility (CARF) at Queensland University of Technology.

### Quality control of Illumina sequence data

High throughput Illumina sequencing produced >257 million raw reads from the four cDNA libraries. Quality of the sequence data was checked first with FastQC (version 0.11.3) software (Andrews, 2010). Trimmomatic software (version 0.36) was used for quality filtering of raw data (Bolger, Lohse & Usadel, 2014), to trim noisy reads (low quality bases) at both ends, reads <36 bases were not considered. Only high quality and filtered reads (Phred Score, *Q* ≥ 30) were used in the subsequent bioinformatics analyses.

### Bioinformatics analyses

All of the quality controlled raw reads from the four cDNA libraries were pooled to make a single reference transcriptome from Bulimba Creek *M. australiense*. *De novo* assembly of high quality reads was performed using the Trinity software package (version 2014-04-13p1) applying perl script (Trinity –seqType fq—JM 200G—left Combined_1.fastq –right Combined_2.fastq –trimmomatic—CPU 12) and default settings (Haas et al., 2013) to obtain contigs. We assessed transcriptome assembly completeness using the CEGMA software package (Parra, Bradman & Korf, 2007). *De novo* assembled contigs were then blasted against the NCBI non-redundant database using BLAST+ (version 2.2.29) with an e value of 1e^−5^ for significant blast hits. The resulting blasted files were loaded into Blast2GO pro software (version 3.0) (Camacho et al., 2009) to map and annotate sequences to determine potential functions of each transcript based on gene ontology (GO) analysis. Following this step, we exported the top hit species distribution chart using the same Blast2Go pro software to check blast hits matching with other species. The annotated contigs were further used in Blast2Go software for InterPro scanning in order to obtain InterProScan IDs. Annotated and InterPro scanned results were then exported and extracted as text files to use for preparing WEGO plots (Web Gene Ontology Annotation Plotting) to categorize gene functions into three different categories (cellular components, molecular functions and biological processes) (Ye et al., 2006). We also used the *de novo* assembled transcripts for differential gene expression analysis that included: mapping of raw reads against the reference transcriptome using Bowtie (version 1.1.1) (Song, Florea & Langmead, 2014), abundance estimation using RSEM (version 1.2.7) (Li & Dewey, 2011) and differential gene expression analysis using edgeR Bioconductor (version 3.3) (Robinson, McCarthy & Smyth, 2010). The RSEM approach generates expression value matrices by calculating maximum likelihood abundance estimation at 95% credibility intervals for genes. Expression value matrices were normalized via the Trimmed Mean of M-values (TMM) method in edgeR to adjust for library size and skewed expression of transcripts (Nakasugi et al., 2013).

### Identification of potential genes

Prior to bioinformatics analysis, a list of candidate genes (Table S1) was prepared from a literature survey. The blasted and annotated transcriptome data sets were mined for candidate gene identification. Results of Blastx searches identified a large number of transcripts belonging to candidate gene families and the full length of each transcript was identified using open reading frames (ORF) finder to identify a start and stop codon in both strands and to determine if each transcript sequence Blastx alignment was used (Liu et al., 2012).

## Results

### Illumina sequencing, *de novo* assembly and annotation

Illumina sequencing results from four different cDNA libraries based on important osmoregulatory tissues (gill, antennal gland, hepatopancreas) as well as a whole individual of post larvae from *M. australiense* are presented in Table 1.

**Table 1:** Summary of raw reads and quality control of Illumina results (*Q* ≥ 30, 75 bp paired-end reads).

Sequencing parameters	Gill	Antennal gland	Hepatopancreas	PL
Number of raw reads	65,713,435	61,417,916	59,472,637	70,538,605
Read length (bp)	26–70	35–76	26–70	26–70
% of GC content	42	42	43	42
Number of read after trimming	61,184,264	57,182,730	55,385,162	65,229,834
% of read after trimming	93.10	93.10	93.12	92.47

Data from the four cDNA libraries were pooled together to make a single reference transcriptome for *M. australiense*. Overall, Illumina paired end sequences were assembled into a total of 125,196 non-redundant contigs (≥200 bp) with an N50 length of 2,282 bp (Table 2). Average and median contig length**s** for the transcriptome were 968 bp and 389 bp respectively. 27,052 contigs were greater than 1,000 bp in length. CEGMA (Core Eukaryotic Genes Mapping Approach) based transcriptome assembly completeness test revealed 97.98% completeness indicating a high quality transcriptome assembly. An overview of assembly results are presented in Table 2.

**Table 2:** Summary of *de novo* assembly and functional annotation results for *M. australiense*.

Number of contigs	125,196
Total bases in contigs	124,731,462
Number of large contigs (≥1,000 bp)	27,052
N50 (bp)	2,282
Average contig length	968
Median contig length	389
Contigs blasted	32,597
Contigs mapped	29,160
Contigs annotated	20,722
Transcriptome assembly completeness	97.98%

**Figure 1:**
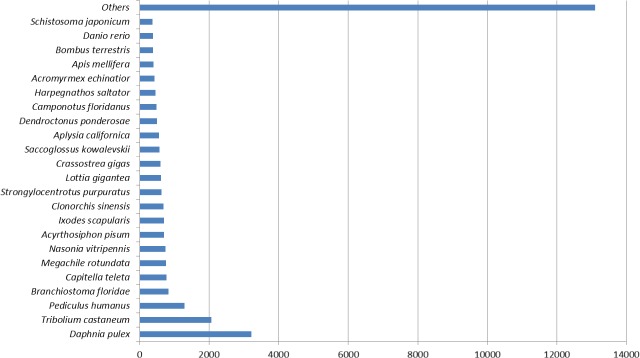
Top hit species distribution chart.

Figure 1 represents the top hit species distribution chart from blast results. *Daphnia pulex* was the top hit species. While *D. pulex* is distantly related to *M. australiense*, it is to date, one of the crustacean species for which a complete sequenced and well annotated genome is available. The ‘Others’ category contained many crustacean taxa including commercially important Penaeids, in particular, *P. monodon* and * L. vannamei*.

**Table 3:** Summary of identified candidate genes potentially associated with osmoregulation in *M. australiense* from this study.

Contig ID	Contig (Gene) name	Contig length (bp)	Amino acid length	Gene Ontology (GO)
c57301_g1_i3	Alkaline phosphatase	2,671	548	Phosphatase activity; metabolic process
c54762_g4_i3	Arginine kinase	1,843	363	ATP binding; kinase activity; phosphorylation
c58410_g1_i4	Aquaporin (AQP)	5,176	299	Substrate-specific transmembrane transporter activity; transmembrane transport
c58153_g1_i1	ATP-binding cassette	11,145	3,471	Protein binding; cellular process; regulation of biological process; response to a stimulus
c52504_g1_i1	Carbonic anhydrase (CA)	3,027	269	Carbonate dehydratase activity; zinc ion binding
c46606_g1_i1	Calreticulin	1,717	404	Calcium ion binding
c37700_g1_i1	H^+^ transporting ATP synthase	944	233	Proton-transporting ATP synthase activity, ATP synthesis coupled proton transport
c59162_g1_i2	Integrin	7,468	1,771	Cell adhesion, stress response
c45418_g1_i2	Interleukin	2,113	538	Cellular response to unfolded protein
c48742_g1_i1	Mitochondrial carrier	2,728	310	Ion transport
c55567_g3_i2	Na^+^/K^+^-ATPase alpha subunit	4,263	1,036	Sodium, potassium-exchanging potassium ion transport; sodium ion transport; cation transmembrane transport
c55187_g2_i1	P38 map kinase	776	103	MAP kinase activity; ATP binding; protein phosphorylation
c46739_g1_i1	V-type proton (H^+^)-ATPase	2,931	426	Proton-transporting V-type ATPase, proton-transporting ATP synthase activity, proton-transporting ATPase activity, ATP hydrolysis coupled proton transport
c55321_g1_i1	USP6 n-terminal-like	2,474	724	Regulation of GTPase activity
c57918_g1_i1	Sodium Hydrogen Exchanger	6,777	672	An integral component of membrane; sodium: proton antiporter activity, sodium ion transport; regulation of pH; inorganic cation transmembrane transport
c57172_g1_i5	Sodium bicarbonate cotransporter	6,754	1,191	Sodium, bicarbonate symporter activity; anion, anion antiporter activity; sodium ion transport; chloride transport; bicarbonate transport
c54650_g1_i1	Sodium potassium chloride cotransporter	4,878	1,051	Sodium, potassium, chloride symporter activity; sodium, chloride symporter activity; amino acid transmembrane transport; potassium ion transport; rubidium ion transport; chloride transmembrane transport
c8687_g1_i1	Selenophosphate (SPS)	2,204	326	Involved in stress tolerance
c88643_g1_i1	Sodium myo-inositol cotransporter	2,453	669	Ion transporter, transmembrane transport
c47111_g1_i1	Sodium channel protein 60e	1,907	385	Regulation of ion transmembrane transport; sodium ion transmembrane transport regulation of ion transmembrane transport
c57445_g1_i2	Sodium Calcium Exchanger 1	8,944	814	Integral component of membrane; calcium, sodium antiporter activity; calcium ion transport; cell communication; transmembrane transport
c52329_g1_i1	Selenoprotein s	2,044	209	Regulation of biological process; regulation of cellular process; response to stimulus
c58145_g1_i4	Potassium voltage-gated channel protein shaker-like isoform 1	5,784	618	Potassium ion transport; transmembrane transport; signal transduction by phosphorylation
c44674_g1_i2	Potassium Sodium hyperpolarization- channel	1,422	342	Voltage-gated potassium channel activity; potassium ion transmembrane transport
c58353_g6_i2	Potassium channel subfamily K member 16-like	2,476	640	Potassium channel activity; potassium ion transmembrane transport
c53215_g1_i2	Mitochondrial transcription factor isoform a	2,244	252	Response to oxidative stress and stress tolerance
c52854_g1_i1	Magnesium transporter protein 1-like	3,159	326	Magnesium ion transmembrane transporter activity
c52684_g1_i1	Heat shock protein 90	3,461	726	Response to stress
c58231_g3_i1	Chorion peroxidase	4,172	922	Response to oxidative stress; oxidation–reduction process
c58101_g3_i4	Chloride channel protein 3	6,326	826	Chloride transport; ion transmembrane transport; regulation of anion transport
c28616_g2_i1	Calcium-binding mitochondrial carrier protein 1	293	697	Transport; biological regulation, regulation of cellular process
c56962_g1_i7	Bestrophin isoform a	2,668	370	Chloride transport; cellular water homeostasis

### Identification of putative osmoregulatory gene families (contigs)

A total of 32 different candidate gene families that are potentially involved with osmoregulation (Table 3) were identified based on blast hits and GO annotation. Most of the key candidate genes involved with osmoregulation in aquatic crustaceans were identified in the analysis of the* M. australiense* transcriptome.

**Figure 2:**
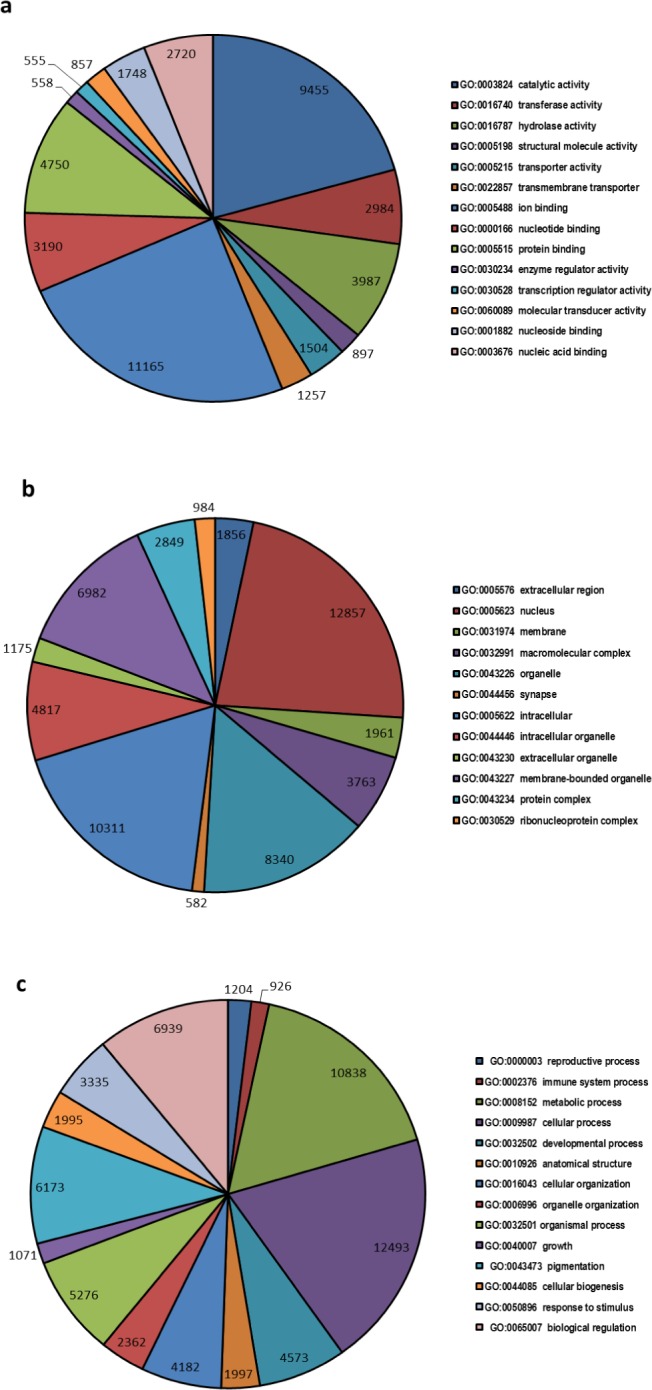
Top most represented GO terms for different categories. Top most abundant GO categories for: (A) molecular function, (B) cellular component and (C) biological process.

A total of 32,597 transcripts were specified with 145,870 GO terms and were assigned to GO categories, as either associated with molecular function (33551 GOs), biological process (73826 GOs) or cellular component (38493 GOs) (Fig. 2). The highest numbers of GO term categories were found to be involved with biological process while molecular function had the smallest number.

### Differential gene expression (DGE) patterns

**Figure 3:**
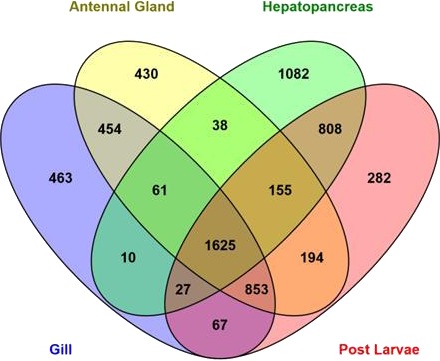
Number of expressed genes in different samples.

Abundance estimation of expressed genes based on transcript per million values (TPM ≥ 20) showed that 3810, 2707, 3806 and 4011 transcripts were expressed in antennal gland, gill, hepatopancreas and post larval tissues respectively (Fig. 3). A total of 1,625 transcripts were found to be in common and expressed in all libraries. Table 4 shows the expression patterns (TPM values) of the top most 10 important candidate osmoregulatory genes. The heatmap (based on quality filtered raw read counts for differentially expressed transcripts) in Fig. S1 shows the qualitative pattern of differentially expressed transcripts in different *M. australiense* tissues.

**Table 4:** Top 10 osmoregulatory genes expressed in different tissues in adult and PL individuals.

Contig ID	Contig description	Transcript Per Million (TPM)			
		Gill	Antennal gland	Hepatopancreas	Post larvae
c54762_g4_i3	Arginine kinase	7,098	5,525	2,077	4,153
c55567_g3_i2	Na^+^/K^+^-ATPase	660	407	90	266
c46606_g1_i1	Calreticulin	385	262	683	524
c57445_g1_i2	Na^+^/Ca^+2^ exchanger	71	74	28	80
c54650_g1_i1	Na^+^/K^+^/2Cl^−^ cotransporter	115	73	50	78
c57172_g1_i5	Na^+^/HCO_3_^-^ cotransporter	61	54	4	25
c46739_g1_i1	V-type ATPase	70	33	70	85
c59162_g1_i2	Integrin	38	31	5	21
c52504_g1_i1	Carbonic anhydrase	211	27	20	41
c8687_g1_i1	Selanophosphate	12	22	7	19

## Discussion

Palaemonid prawns contain the most impressive list of crustacean invaders of freshwater from an ancestral marine habitat and they manifest a diverse array of osmoregulatoy capacities (Towle, Henry & Terwilliger, 2011). Various tissues including gill, antennal gland and hepatopancreas all contribute to ion balance and regulation that has allowed this family to colonise a wide variety of osmotic aquatic niches (Shekhar, Kiruthika & Ponniah, 2013). New technologies linked to NGS developments have allowed mechanisms that permit adaptive colonisation of freshwater to be explored with high data accuracy, at relatively low cost and generation of very large amounts of data (Lv et al., 2013). The current study generated a comprehensive NGS database for a freshwater-adapted Palaemonid species as an initial step to better understand the role of various potential candidate genes to be identified with a function in osmoregulation performance and to provide a model for more detailed investigations of other related and commercially important crustacean taxa. The transcriptome was generated as a starting point to investigate the molecular foundation for osmoregulation in FW crustaceans. *M. australiense* is a freshwater inhabitant, but this taxon’s ability to tolerate brackish water conditions is well documented (Sharma & Hughes, 2009). This species is widely distributed and abundant across Australia and possesses a comparatively large body (provides sufficient tissue material) size, characteristics that make it an ideal candidate species for identification of the molecular basis of osmoregulation (and the associated candidate genes involved) under different salinity conditions. The highest number of raw reads (over 70 million) were obtained for post larvae (Table 1) because at this early stage of the life cycle, a higher number of genes are expressed. Different genes are expressed in different tissue types and more reads were obtained from tissues where more genes were actively expressed (Tian et al., 2011). Thus, availability of reads can vary according to the tissue sampled. The transcriptome data set generated here from four cDNA libraries resulted over 250 million copies of 75 bp paired end raw reads that generated 125,196 longer contigs from *de novo* assembly. In total, 32 important gene families were identified that have potential roles in osmoregulation and ion regulation that contribute to salinity tolerance in this species. Overall, 32,597 (25.43%) of the assembled contigs showed significant blast hits and 20,722 (16.55%) contigs produced GO term annotations (Table 2). In the current study, we identified more potential osmoregulatory genes (32 genes, Table 3) compared with the total number of genes (21 genes, Table S1) identified in the literature survey. This is because we focused only on prawn species for the literature review but our GO annotation approach involved blast matching with all identified genes for many different species that were available in the public database. Thus, the *M. australiense* transcriptome dataset provided us with a set of potential target genes available (not only for osmoregulation but for many other phenotypes) for further explanation in this and other related species with different osmotic capacities so that an investigation of the key genes and their functional mutations can be explored in greater details.

Abundance estimates of expressed genes based on TPM values showed that 1,625 transcripts common to, and expressed in, all four libraries that represent different life history stages and tissue types. The 1,625 common transcripts (Fig. 3) suggest an important functional role (potentially housekeeping roles) for basic cellular maintenance in this species as they are expressed in all tissues and at all different stages of life. The hepatopancreas in adults showed the highest number of distinct expressed transcripts (1,082) implying that it is probably the most important tissue as this is the master tissue that controls most body functions. A higher number of transcripts (808) were found to be expressed commonly between hepatopancreas and in PLs indicating expression of important genes that are required for all stages of life. Gill and antennal gland tissues shared 454 transcripts in common. This is likely because these two tissues are involved with ion exchange (osmoregulatory function), gas exchange and some other common functions (Lignot, Spanings-Pierrot & Charmantier, 2000). Based on identified candidate genes in other species, we were able to target the most important genes related to metabolic process, ATP binding, transmembrane activities, regulation of ion transportation, response to environmental stresses and stress tolerance in *M. australiense*. Efficient osmoregulatory capacity does not depend on ion exchange only; it also involves many other biological processes. Of the 32 osmoregulatory gene families identified here, 17 genes were identified to be involved with transport of the most important ions (Na^+^, K^+^, H^+^, Ca^2+^, Mg^2+^, HCO${}_{3}^{-}$ and Cl^−^) all of which impact osmoregulation and influence salinity tolerance. Differential expression patterns (based on transcript abundance estimates) of potential candidate genes involved with osmoregulation, clearly indicate the more important role of gill and antennal gland tissues to perform osmoregulation in adult individuals as the expression patterns of potential osmoregulatory genes in these tissues were high compared with that seen in the hepatopancreas (Table 4). Of interest however, was that only post larvae showed moderate expression patterns for the same candidate genes. Differential gene expression patterns showed a higher number of genes expressed differentially in post larvae (Fig. S1). This is likely related to rapid developmental changes that are occurring during earlier life history stages that require raised levels of expression of genes involved with growth, tissue development and other developmental processes. The heatmap also shows the highest level of expression pattern in hepatopancreas for adult prawns because this tissue is considered to be the master organ that controls most of cellular activity.

Arginine kinase (AK) in gill tissue has been recognised as one of the most important genes associated with osmoregulatory performance. Expression levels of AK in crustacean gill tissue is generally very high in actively osmoregulating individuals (in energy production and metabolism and of amino acid sequence) (Abe, Hirai & Okada, 2007; Kotlyar et al., 2000), but it also has a role in immune responses (Kinsey & Lee, 2003; Yao et al., 2009). In the current study, AK levels in the four *M. australiense* cDNA libraries were highest in gill tissue (7,098 expressed copies, Table 4) indicating that it may also function in energy generation for osmoregulation in *M. australiense* as well.

High expression levels of certain other genes including the calreticulin in the hepatopancreas and also in PL indicates that this gene family has a role in many biological processes ranging from Ca^2+^ storage (more important for PL stage involved in calcifications and metamorphosis) (Barman et al., 2012; Xu et al., 2015), gene expression regulation (Duan et al., 2014; Gelebart, Opas & Michalak, 2005), signalling pathway and fast immune reaction against serious environmental stressors other than osmotic regulation (Tian et al., 2011; Visudtiphole et al., 2010).

NKA (Na^+^/K^+^-ATPase), is also critical for osmoregulation in* M. australiense*. This gene is also an important integral membrane protein involved in ion transportation and ammonia excretion, so it has an associated role in osmoregulatory systems in many taxa, but particularly in aquatic crustacean species (Tsai & Lin, 2007). The activity of NKA in osmoregulatory tissues, where it plays a major role in ion exchange, has been investigated in both hyper/hypo osmoregulating and osmoconforming crustacean taxa (Charmantier & Anger, 2011; Cieluch et al., 2004; Lucu & Towle, 2003). In this study, we also identified different NKA isoforms and subunits of NKA ranging from 819–4,263 bp in sequence length. Maximum expression level was evident in gill tissue where gills obviously provide the major osmoregulatory organs (Table 4). NKCC (Na^+^/K^+^/2Cl^−^ cotransporter) located in the basal membrane drives Cl^−^ and Na^+^ exclusion across salt-secreting epithelia and is involved in both Na^+^ and Cl^−^ uptake and excretion powered by Na^+^ and Cl^−^ turnover. Mediation of relative expression levels of NKA during hypo/hyper osmotic challenges (Luquet et al., 2005) has been demonstrated in a number of studies, in particular two different crab taxa *Chasmagnatus granulates* and *Carcinus maenas* (Genovese et al., 2000). Here we also found 212–4,878 bp of the NKCC gene family involved in Na^+^, K^+^ and Cl^−^ transport activity and transmembrane transport.

The different functions of Carbonic Anhydrase (CA) in cellular components, respiration and metabolic process have been the focus of many case studies. Its pivotal role in the crustacean gills is associated with Na^+^ and Cl^−^ regulation and directly is involved in ion transport that also was identified in the * M. australiense* transcriptome. Transcript lengths ranged from 218–3,027 bp. This enzyme constitutes one of the primary components of the osmotic regulation, is highly expressed in the organs that are responsible for osmotic and ionic regulation (Jasmani et al., 2010; Jasmani, Jayasankar & Wilder, 2008; Roy et al., 2007).

Another significant gene involved with osmoregulation in crustaceans is V-type ATPase (VA) that is a crucial gene for regulation of ion balance and cell volume (Freire, Onken & Mcnamara, 2008). The important role/s of this gene have been documented in many aquatic animals including in; euryhaline crabs, *Carcinus maenas* and *Eryocheir sinensis* (Weihrauch et al., 2001) and two freshwater crayfish, *Cherax destructor* and *C. cainii* (Ali et al., 2015). Here, we identified an *M. australiense* VA gene and the recovered gene ontology terms indicate a role for this gene in many different complex mechanisms including acid/base balance, nitrogen excretion, ion exchange and proton regulation in the freshwater environment.

Some other candidate genes identified here were found to be involved with cellular response to stress, ATP binding, regulation of biological responses, response to stimuli and in oxidative stress. Potential genes for these processes likely include: selenoprotein, aquaporin, USP, ATP-binding cassette, alkaline phosphatase (AP), integrin and interleukin (Tables 3 and 4). Among these gene families, AP codes for well-known metallo-enzymes in mammals with diverse functions, mostly related to metabolic processes. Some studies however, have reported a role for this gene in osmoregulatory performance in blue crab, *Callinectess apidus* (Lovett, Towle & Faris, 1994), green crab, *Scylla serrata* (Park et al., 2001) and in the euryhaline crab, *Cyrtograpsus angalatus* (Pinoni and Manñanes, 2004). Aquaporin acts as a water channel for selective transfer of water and other molecules across cell membranes in gill and antennal gland tissues (Gao, 2009). This gene is directly involved in cellular osmotic challenges and cell volume regulation. It also is one of the most-studied genes across taxa (invertebrates to mammals) because it has a reported functional role in salinity tolerance (Barman et al., 2012; Boyle et al., 2013; Grosell, 2006; Nishimura & Fan, 2003; Pongsomboon et al., 2009).

In summary, the current study has generated a comprehensive genomic resource for identification of key candidate genes involved in osmoregulation in *M. australiense* and has also provided a platform for functional genomic studies of other freshwater prawn taxa more widely. 32 different potential genes were identified that are likely to play major functional roles in osmoregulation and salinity tolerance processes. The current study can provide a baseline for developing a more comprehensive understanding of the functional genomic basis of ion exchange and ion balance processes in invertebrate aquatic taxa. Results of this study could be used for functional genomic scans among different populations of the target species collected from different natural osmotic niches to better understand the functional genomic adaptive processes occurring in different natural populations. In the future, it will also be important to assess the gene expression patterns of the major candidate genes identified here under different salinity conditions in *M. australiense* to explore the interactions that contribute to effective ionic regulation in this species.

##  Supplemental Information

10.7717/peerj.2520/supp-1 Table S1 Specific genes previously identified as having a functional role in salinity tolerance in crustaceans

10.7717/peerj.2520/supp-2 Figure S1 Heatmap showing differential gene expression pattern (based on read counts) of different tissues of adult and post larvae. AEB, Antennal Gland, GB, Gill; HIB, Hepatopancreas; PL, Post Larvae. *Y*-axis represents the hierarchical clus

10.7717/peerj.2520/supp-3 Supplemental Information 1 Bioanalyzer Report for total RNA Quality and Concentration

## Additional Information and Declarations

### Field Study Permissions

The following information was supplied relating to field study approvals (i.e., approving body and any reference numbers): The field permit was issued by the Department of Agriculture, Fisheries and Forestry under Queensland Government. Permit Number: 166312.

### Data Availability

The following information was supplied regarding data availability: Assembly: Moshtaghi, Azam; Rahi, Lifat (2016): Macrobrachium australiense Assembly. Figshare.
https://dx.doi.org/10.6084/m9.figshare.3444992.v1. Raw Illumina Sequence Data: Moshtaghi, Azam; Rahi, Lifat (2016): Forward Sequences for Antennal Gland. Figshare.
https://dx.doi.org/10.6084/m9.figshare.3628464.v1. Moshtaghi, Azam; Rahi, Lifat (2016): Reverse Sequences for Antennal Gland. Figshare.
https://dx.doi.org/10.6084/m9.figshare.3628665.v1. Rahi, Lifat; Moshtaghi, Azam (2016): Forward Sequences for Gill. Figshare.
https://dx.doi.org/10.6084/m9.figshare.3627861.v1. Rahi, Lifat; Moshtaghi, Azam (2016): Reverse Sequences for Gill. Figshare.
https://dx.doi.org/10.6084/m9.figshare.3628113.v1. Moshtaghi, Azam; Rahi, Lifat (2016): Forward Sequences for Hepatopancreas. Figshare.
https://dx.doi.org/10.6084/m9.figshare.3628869.v1. Moshtaghi, Azam; Rahi, Lifat (2016): Reverse Sequences for Hepatopancreas. Figshare.
https://dx.doi.org/10.6084/m9.figshare.3629055.v1. Rahi, Lifat; Moshtaghi, Azam (2016): Forward Raw Reads for PL. Figshare.
https://dx.doi.org/10.6084/m9.figshare.3626667.v1. Rahi, Lifat; Moshtaghi, Azam (2016): Reverse Raw Reads for PL. Figshare.
https://dx.doi.org/10.6084/m9.figshare.3627504.v1. CO1 Sequences: Moshtaghi, Azam (2016): CO1 Gene Sequences. Figshare. https://dx.doi.org/10.6084/m9.figshare.3625773.v1.
