# Supplementary material for: A transcriptomic scan for potential candidate genes involved in osmoregulation in an obligate freshwater palaemonid prawn (Macrobrachium australiense)
Source: PeerJ. 2016 Oct 5;4:e2520. doi: 10.7717/peerj.2520 (PMC5068373; doi:10.7717/peerj.2520)
Supplement: Table S1 [file peerj-04-2520-s001.docx]

**Supplemental Table**

| **GENE** | **FUNCTION** | **REFERENCE** |
| --- | --- | --- |
| Aquaporin 3 (AQP) | cell volume regulation and water permeability under stress | ([Gao, 2009](#_ENREF_19)) |
| Calreticulin (CRT) | signal transduction in many biological processes of growth, moulting and stress response | ([Luana et al., 2007](#_ENREF_41)) |
| Na^+^/K^+^- ATPase (NKA) | creating an electrochemical gradient across the cell membrane in the gills of crustaceans for actively absorb or release ions | ([Santos et al., 2007](#_ENREF_55))  ([Boudour-Boucheker et al., 2014](#_ENREF_8)) |
| Na^+^/K^+^/2Cl^-^ cotransporter (NKCC) | transport ions into gill cells either from the blood or media based on salinity | ([Faleiros et al., 2010](#_ENREF_17)) |
| Carbonic anhydrase (CA) | involved in all main gill function such as regulation of Na^+^, Cl^-^, H^+^, HCO3^-^ (specifically in low salinities) | ([Henry et al., 2003](#_ENREF_27)) |
| V type- (H^+^) ATPase | role in salinity acclimation and osmoregulation | ([Tsai and Lin, 2007](#_ENREF_62)) |
| ILF2 (interlukin enhancer binding factor 2) | a transcriptional regulator against biotic stresses | ([Barman et al., 2012](#_ENREF_5)) |
| Alkaline phosphatase | involved in salinity acclimation and osmoregulation response | ([Lovett et al., 1994](#_ENREF_40)) |
| Selenophosphate (SPS1) | involved in salinity tolerance, oxidative role | ([Gillanders et al., 2003](#_ENREF_22)) |
| Integrin | involved in salinity stress | ([Pongsomboon et al., 2009](#_ENREF_51)) |
| ABC protein C12 (ABCC 12) | functional role in osmoregulation and salinity tolerance under osmotic stresses | ([Liu et al., 2012](#_ENREF_39)) |
| P38 MAP kinase | regulation processes (mRNA stability, protein degradation) under environmental stress | ([He et al., 2013](#_ENREF_25)) |
| USP | involved in salinity tolerance | ([Kotlyar et al., 2000](#_ENREF_36)) |
| Na^+^/H^-^exchanger | regulation of Na^+^ and H^-^ levels and involved in regulation of extracellular acid-base | ([Freire et al., 2008](#_ENREF_18)) |
| Ca^2+^ -ATPase | involved in Ca^2+^ regulation and calcification | ([Freire et al., 2008](#_ENREF_18)) |
| Cystic Fibrosis Transmembrane Regulator (CFTR) | regulation of Cl^-^ channels and increases the osmoregulatory capacity | ([Bodinier et al., 2009](#_ENREF_6)) |
| Arginine kinase | involved in active ion transport across membrane | ([Kotlyar et al., 2000](#_ENREF_36)) |
| OST complex | maintaining homeostasis during salinity/osmotic stress | ([Pongsomboon et al., 2009](#_ENREF_51)) |
| Acyl-CoA binding protein (ACBP) | role in salinity tolerance and adaptation within low and high salinity levels | ([Kiruthika et al., 2013](#_ENREF_35)) |
| Catechol-o-methyltransferase (COMT) | association with low and high salinity and crucial role within salinity stresses | ([Rajesh et al., 2012](#_ENREF_52)) |
| Mitochondrial carrier protein | involved in osmoregulation signal transduction | ([Menze et al., 2005](#_ENREF_46)) |

**Table S1:** Specific genes previously identified as having a functional role in salinity tolerance in crustaceans.
