# Supplementary material for: A transcriptomic scan for potential candidate genes involved in osmoregulation in an obligate freshwater palaemonid prawn (Macrobrachium australiense)
Source: PeerJ. 2016 Oct 5;4:e2520. doi: 10.7717/peerj.2520 (PMC5068373; doi:10.7717/peerj.2520)
Supplement: Figure S1 [file peerj-04-2520-s002.pdf]

Supplemental Figure

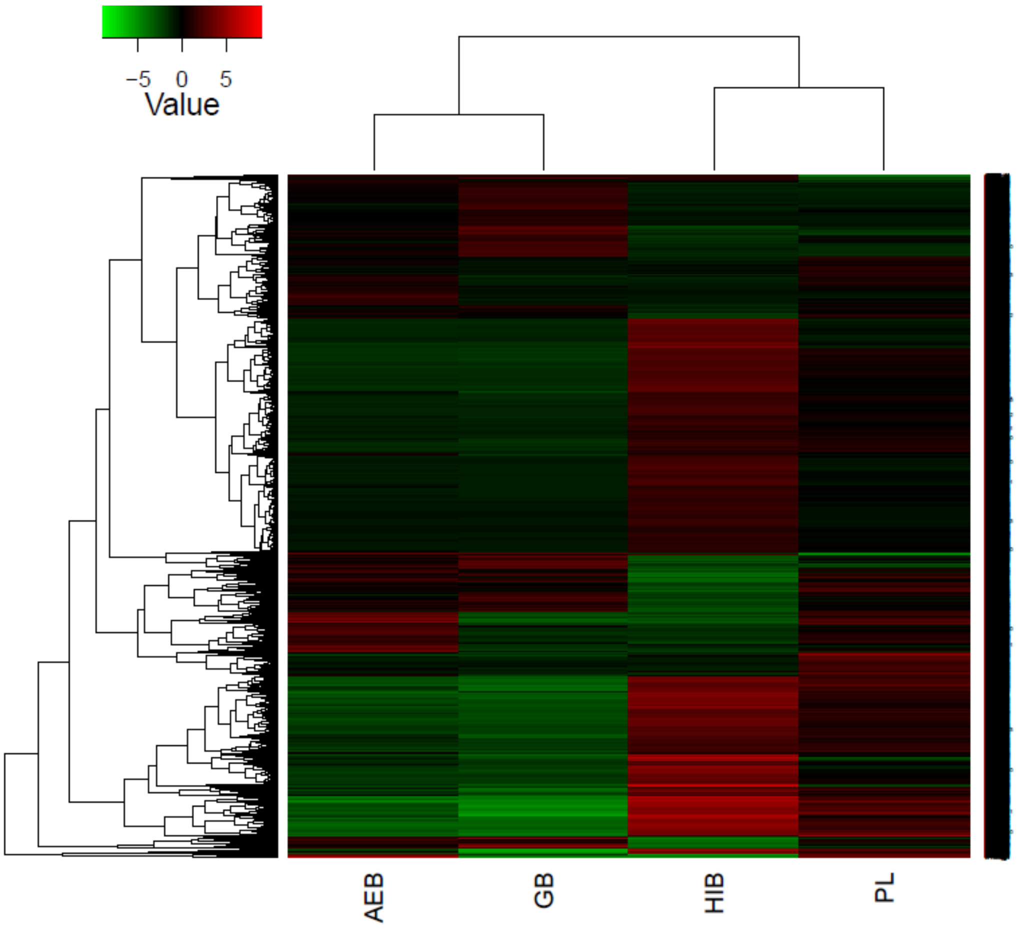

Supplemental Figure S1: Heatmap showing differential gene expression pattern (based on read counts) of different tissues of adult and post larvae. AEB= Antennal Gland, GB= Gill, HIB= Hepatopancreas, PL= Post Larvae. **Y-axis represents the hierarchical clustering of differentially expressed transcripts, while red colored lines indicate highly expressed transcripts and green colored lines indicate lowly expressed transcripts.**
