# Supplementary material for: A transcriptomic scan for potential candidate genes involved in osmoregulation in an obligate freshwater palaemonid prawn (Macrobrachium australiense)
Source: PeerJ. 2016 Oct 5;4:e2520. doi: 10.7717/peerj.2520 (PMC5068373; doi:10.7717/peerj.2520)
Supplement: Supplemental Information 1 [file peerj-04-2520-s003.pdf]

Assay Class: Eukaryote Total RNA Nano
Data Path: C:\...Eukaryote Total RNA Nano_DE13804083_2014-04-15_13-05-22.xad

Created: 15/04/2014 1:05:21 PM
Modified: 15/04/2014 1:29:08 PM

Electrophoresis File Run Summary

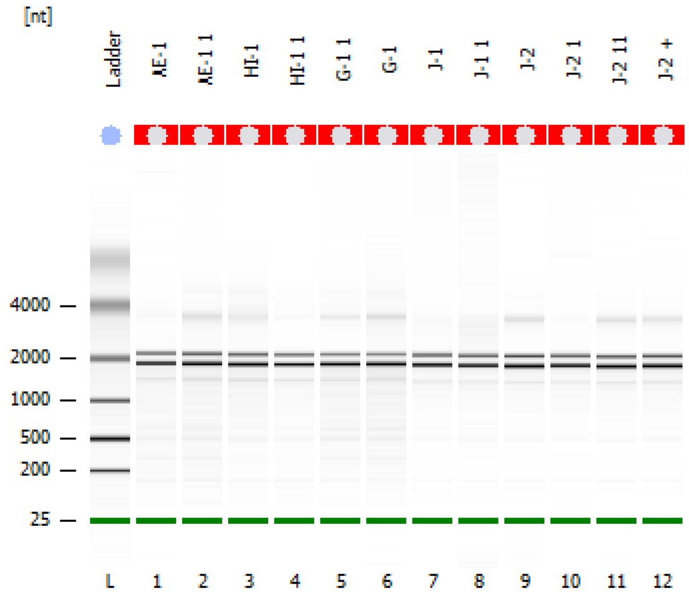

Instrument Information:

Instrument Name: DE13804083
Serial#: DE13804083
Firmware: C.01.069
Type: G2939A

Assay Information:

Assay Origin Path: C:\Program Files (x86)\Agilent\2100 bioanalyzer\2100 expert\assays\RNA\Eukaryote Total RNA Nano Series II.xsy

Assay Class: Eukaryote Total RNA Nano

Version: 2.6

Assay Comments: Total RNA Analysis ng sensitivity (Eukaryote)

© Copyright 2003 - 2009 Agilent Technologies, Inc.

Chip Information:

Chip Lot #:

Reagent Kit Lot #:

Chip Comments:

AE-1

RIN N/A

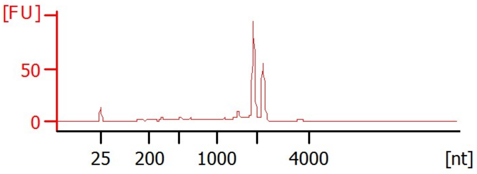

AE-1 1

RIN N/A

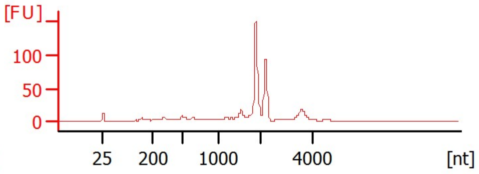

HI-1

RIN N/A

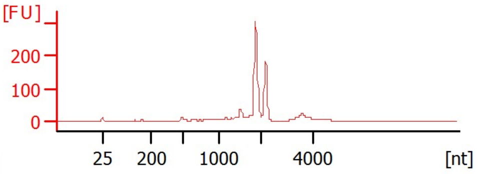

HI-1 1

RIN N/A

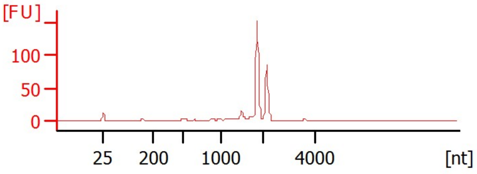

G-1 1

RIN N/A

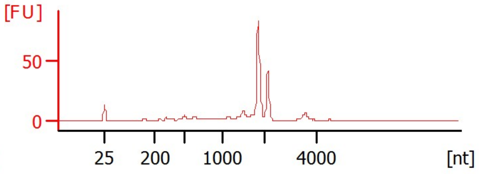

G-1

RIN N/A

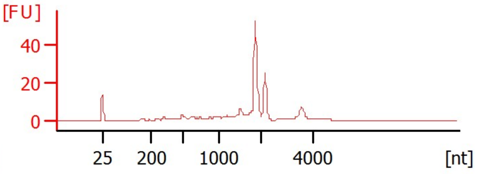

J-1

RIN N/A

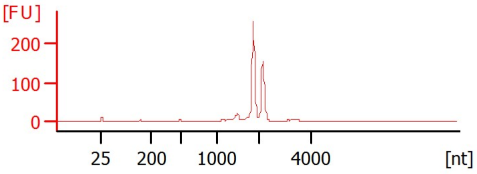

J-1 1

RIN N/A

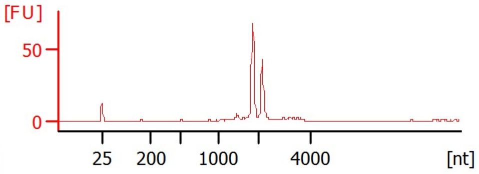

J-2

RIN N/A

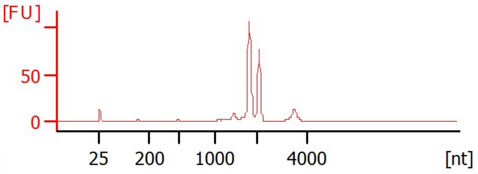

J-2 1

RIN N/A

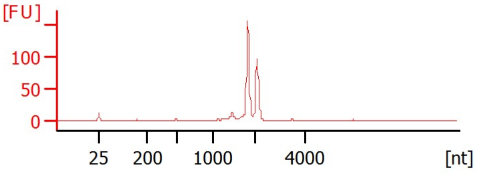

J-2 11

RIN N/A

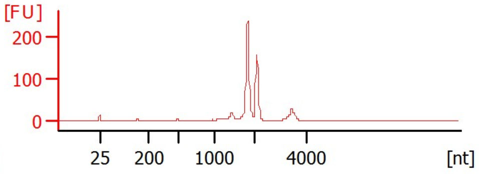

J-2 +

RIN N/A

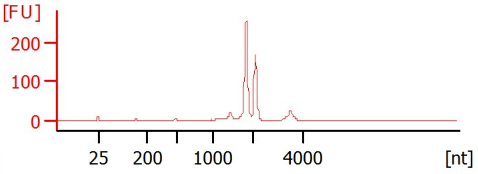

 © Copyright 2003 - 2009 Agilent Technologies, Inc.

Assay Class: Eukaryote Total RNA Nano
Data Path: C:\...Eukaryote Total RNA Nano_DE13804083_2014-04-15_13-05-22.xad

Created: 15/04/2014 1:05:21 PM
Modified: 15/04/2014 1:29:08 PM

Electrophoresis File Run Summary (Chip Summary)

Sample Name	Sample Comment	Status	Result Label	Result Color
AE-1		✔	RIN N/A	
AE-1 1		✔	RIN N/A	
HI-1		✔	RIN N/A	
HI-1 1		✔	RIN N/A	
G-1 1		✔	RIN N/A	
G-1		✔	RIN N/A	
J-1		✔	RIN N/A	
J-1 1		✔	RIN N/A	
J-2		✔	RIN N/A	
J-2 1		✔	RIN N/A	
J-2 11		✔	RIN N/A	
J-2 +		✔	RIN N/A	
Ladder		✔	All Other Samples	

Chip Lot # **Reagent Kit Lot #**

Chip Comments :

 © Copyright 2003 - 2009 Agilent Technologies, Inc.

Assay Class: Eukaryote Total RNA Nano
Data Path: C:\...Eukaryote Total RNA Nano_DE13804083_2014-04-15_13-05-22.xad
Created: 15/04/2014 1:05:21 PM
Modified: 15/04/2014 1:29:08 PM

Electrophoresis Assay Details

General Analysis Settings

Number of Available Sample and Ladder Wells (Max.) : 13
Minimum Visible Range [s] : 17
Maximum Visible Range [s] : 70
Start Analysis Time Range [s] : 19
End Analysis Time Range [s] : 69
Ladder Concentration [ng/µl] : 150
Lower Marker Concentration [ng/µl] : 0
Upper Marker Concentration [ng/µl] : 0
Used Lower Marker for Quantitation
Standard Curve Fit is Logarithmic
Show Data Aligned to Lower Marker

Integrator Settings

Integration Start Time [s] : 19
Integration End Time [s] : 69
Slope Threshold : 0.6
Height Threshold [FU] : 0.5
Area Threshold : 0.2
Width Threshold [s] : 0.5
Baseline Plateau [s] : 6

Filter Settings

Filter Width [s] : 0.5
Polynomial Order : 4

Ladder

Ladder Peak	Size
1	25
2	200
3	500
4	1000
5	2000
6	4000

 © Copyright 2003 - 2009 Agilent Technologies, Inc.

Assay Class: Eukaryote Total RNA Nano
Data Path: C:\...Eukaryote Total RNA Nano_DE13804083_2014-04-15_13-05-22.xad

Created: 15/04/2014 1:05:21 PM
Modified: 15/04/2014 1:29:08 PM

Electropherogram Summary

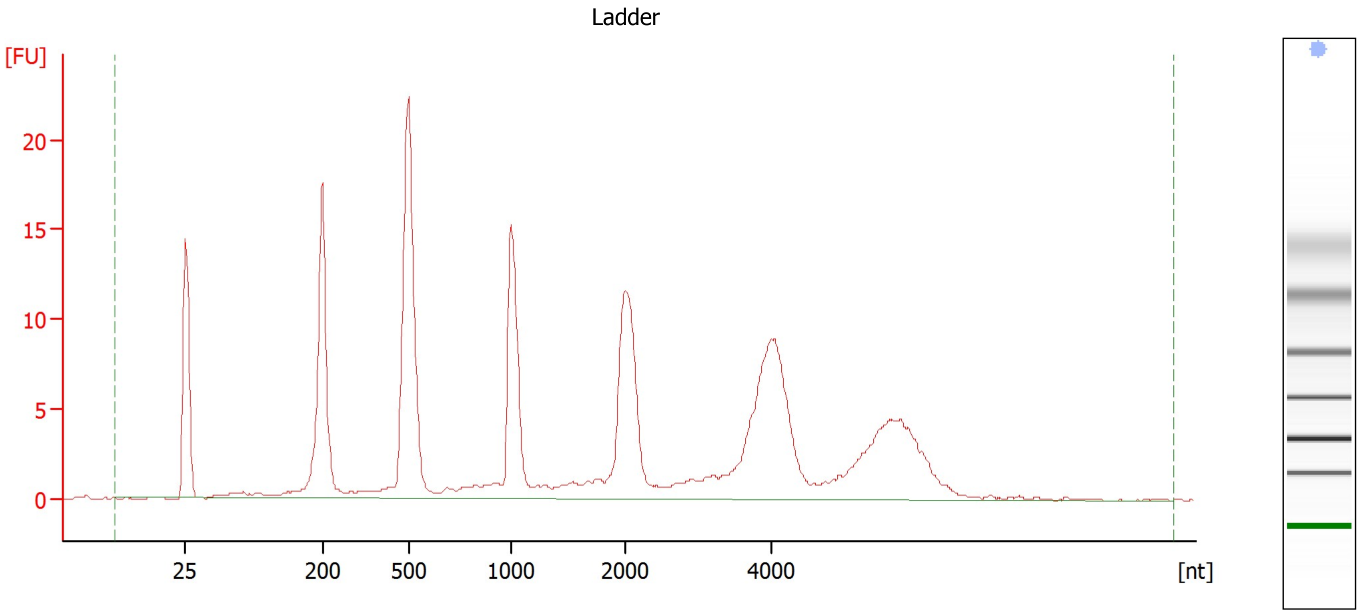

Overall Results for Ladder

RNA Area:	207.2	Result Flagging Color:	
RNA Concentration:	150 ng/µl	Result Flagging Label:	All Other Samples

© Copyright 2003 - 2009 Agilent Technologies, Inc.

Assay Class: Eukaryote Total RNA Nano
Data Path: C:\...Eukaryote Total RNA Nano_DE13804083_2014-04-15_13-05-22.xad

Created: 15/04/2014 1:05:21 PM
Modified: 15/04/2014 1:29:08 PM

Electropherogram Summary Continued ...

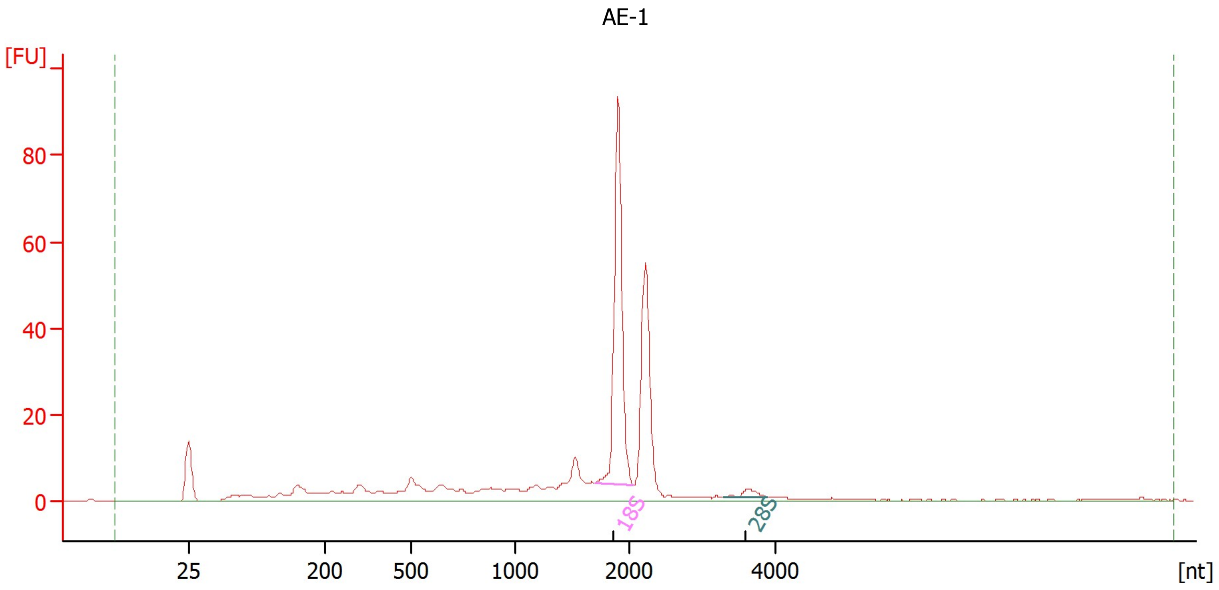

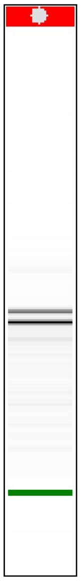

Overall Results for sample 1 : AE-1

RNA Area:	310.2	RNA Integrity Number (RIN):	N/A (B.02.08)
RNA Concentration:	225 ng/µl	Result Flagging Color:	
rRNA Ratio [28s / 18s]:	0.0	Result Flagging Label:	RIN N/A

Fragment table for sample 1 : AE-1

Name	Start Size [nt]	End Size [nt]	Area	% of total Area
18S	1,697	2,046	86.1	27.8
28S	3,276	3,876	3.1	1.0

© Copyright 2003 - 2009 Agilent Technologies, Inc.

Assay Class:	Eukaryote Total RNA Nano	Created:	15/04/2014 1:05:21 PM
Data Path:	C:\...Eukaryote Total RNA Nano_DE13804083_2014-04-15_13-05-22.xad	Modified:	15/04/2014 1:29:08 PM

Electropherogram Summary Continued ...

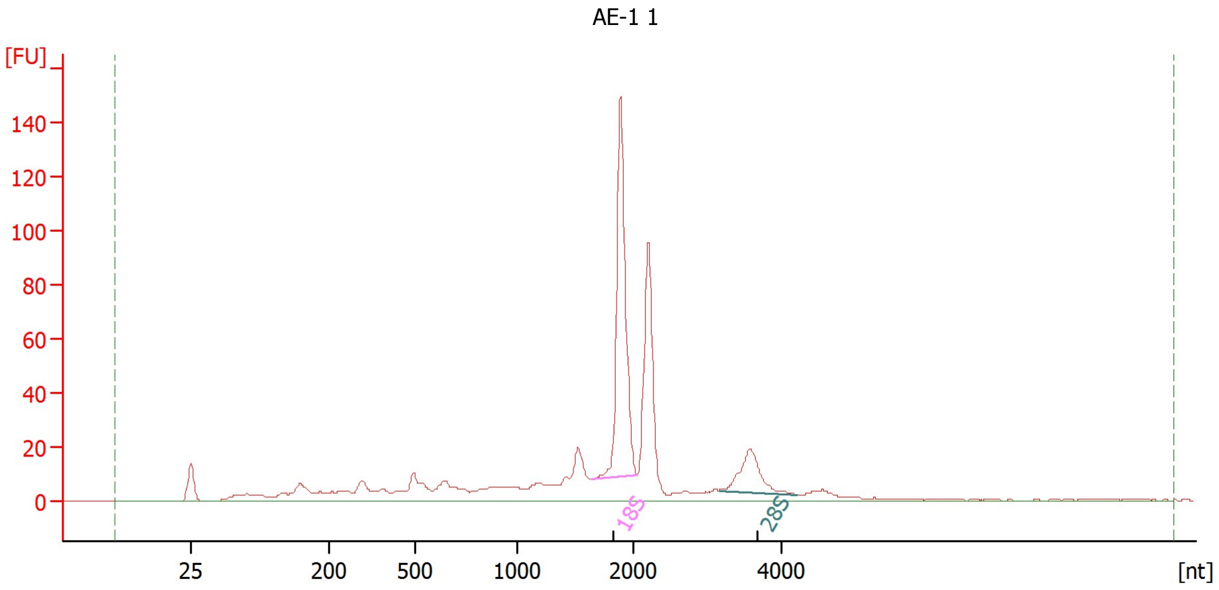

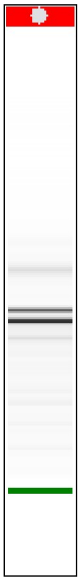

Overall Results for sample 2 : AE-1 1

RNA Area:	638.7	RNA Integrity Number (RIN):	N/A (B.02.08)
RNA Concentration:	462 ng/µl	Result Flagging Color:	
rRNA Ratio [28s / 18s]:	0.2	Result Flagging Label:	RIN N/A

Fragment table for sample 2 : AE-1 1

Name	Start Size [nt]	End Size [nt]	Area	% of total Area
18S	1,636	2,034	153.9	24.1
28S	3,157	4,221	34.5	5.4

 © Copyright 2003 - 2009 Agilent Technologies, Inc.

Assay Class:	Eukaryote Total RNA Nano	Created:	15/04/2014 1:05:21 PM
Data Path:	C:\...Eukaryote Total RNA Nano_DE13804083_2014-04-15_13-05-22.xad	Modified:	15/04/2014 1:29:08 PM

Electropherogram Summary Continued ...

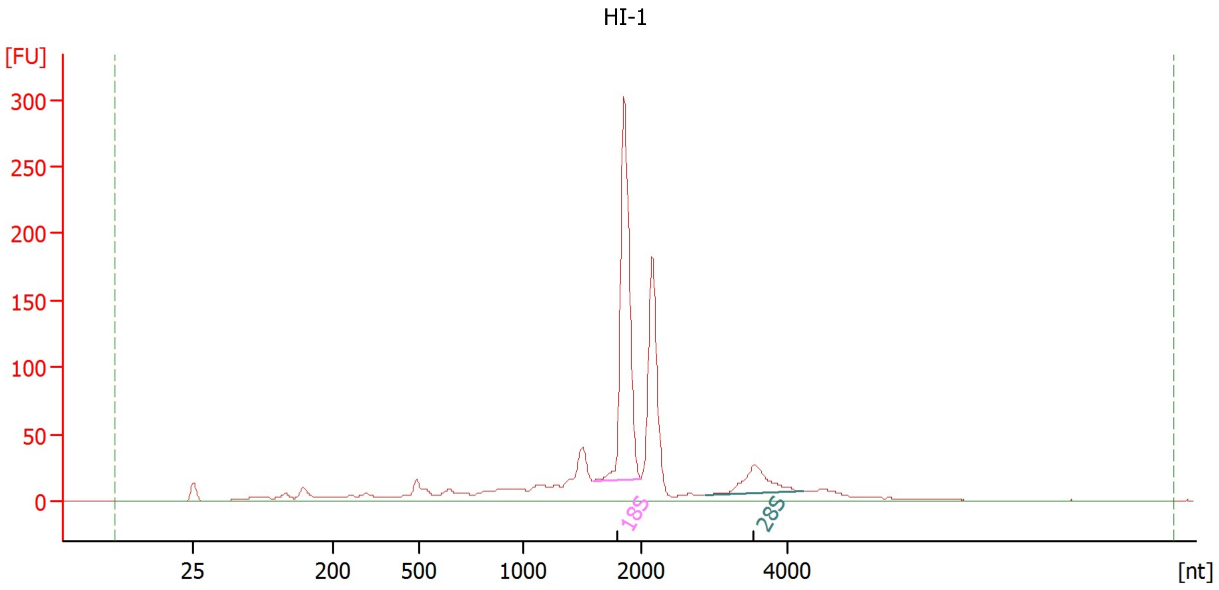

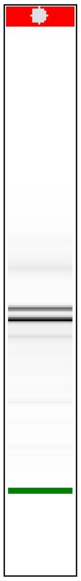

Overall Results for sample 3 : HI-1

RNA Area:	1,113.8	RNA Integrity Number (RIN):	N/A (B.02.08)
RNA Concentration:	806 ng/µl	Result Flagging Color:	
rRNA Ratio [28s / 18s]:	0.2	Result Flagging Label:	RIN N/A

Fragment table for sample 3 : HI-1

Name	Start Size [nt]	End Size [nt]	Area	% of total Area
18S	1,604	2,009	313.7	28.2
28S	2,878	4,211	57.0	5.1

 © Copyright 2003 - 2009 Agilent Technologies, Inc.

Assay Class: Eukaryote Total RNA Nano
Data Path: C:\...Eukaryote Total RNA Nano_DE13804083_2014-04-15_13-05-22.xad

Created: 15/04/2014 1:05:21 PM
Modified: 15/04/2014 1:29:08 PM

Electropherogram Summary Continued ...

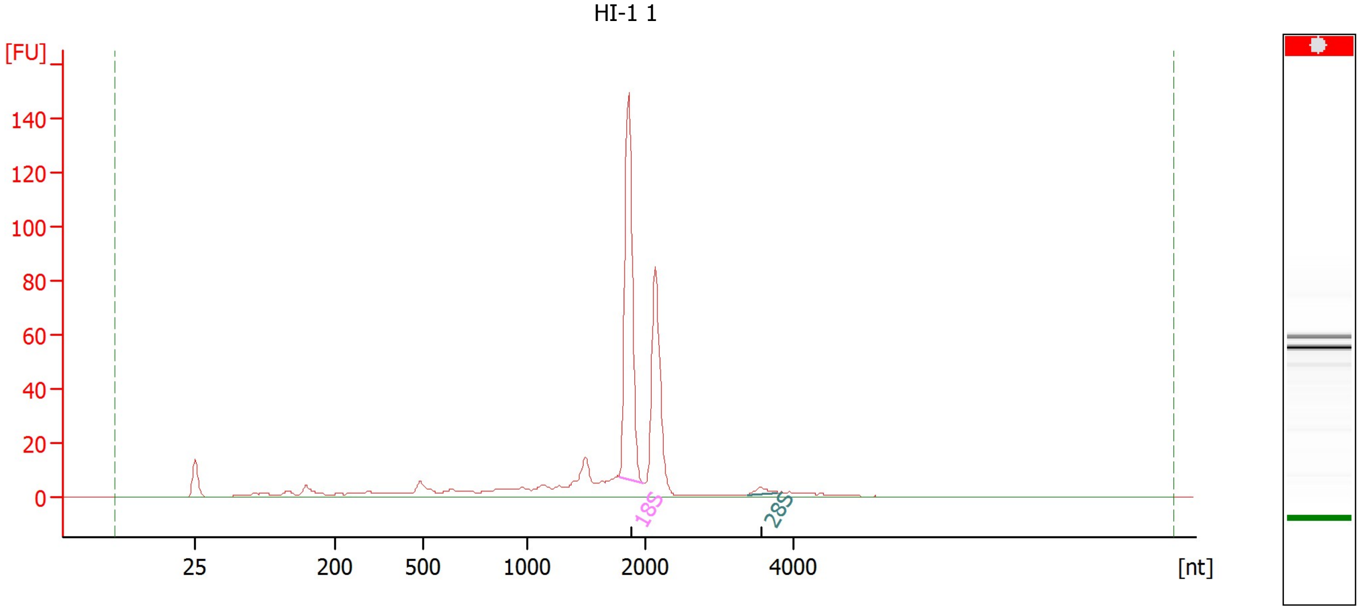

Overall Results for sample 4 : HI-1 1

RNA Area:	394.3	RNA Integrity Number (RIN):	N/A (B.02.08)
RNA Concentration:	286 ng/µl	Result Flagging Color:	
rRNA Ratio [28s / 18s]:	0.0	Result Flagging Label:	RIN N/A

Fragment table for sample 4 : HI-1 1

Name	Start Size [nt]	End Size [nt]	Area	% of total Area
18S	1,780	1,989	130.5	33.1
28S	3,382	3,800	2.9	0.7

 © Copyright 2003 - 2009 Agilent Technologies, Inc.

Assay Class: Eukaryote Total RNA Nano
Data Path: C:\...Eukaryote Total RNA Nano_DE13804083_2014-04-15_13-05-22.xad

Created: 15/04/2014 1:05:21 PM
Modified: 15/04/2014 1:29:08 PM

Electropherogram Summary Continued ...

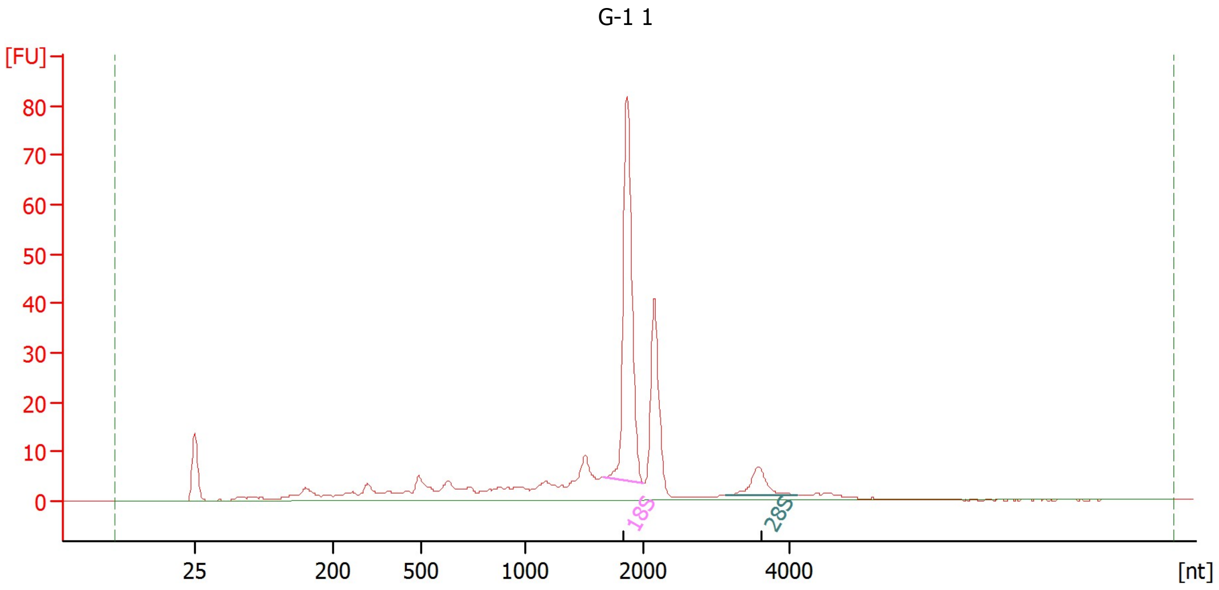

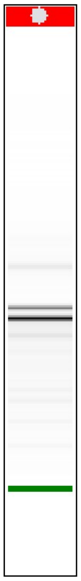

Overall Results for sample 5 : G-1 1

RNA Area:	281.9	RNA Integrity Number (RIN):	N/A (B.02.08)
RNA Concentration:	204 ng/µl	Result Flagging Color:	
rRNA Ratio [28s / 18s]:	0.1	Result Flagging Label:	RIN N/A

Fragment table for sample 5 : G-1 1

Name	Start Size [nt]	End Size [nt]	Area	% of total Area
18S	1,670	2,011	81.5	28.9
28S	3,123	4,106	10.4	3.7

© Copyright 2003 - 2009 Agilent Technologies, Inc.

Assay Class: Eukaryote Total RNA Nano
Data Path: C:\...Eukaryote Total RNA Nano_DE13804083_2014-04-15_13-05-22.xad
Created: 15/04/2014 1:05:21 PM
Modified: 15/04/2014 1:29:08 PM

Electropherogram Summary Continued ...

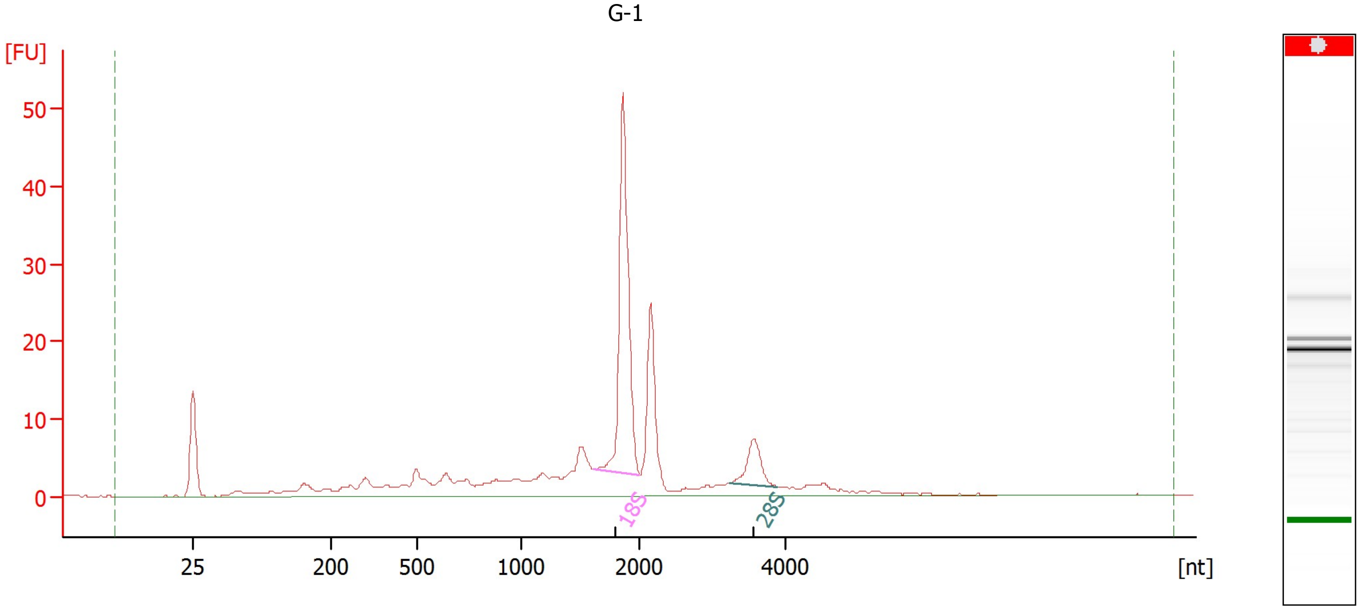

Overall Results for sample 6 : G-1

RNA Area:	221.1	RNA Integrity Number (RIN):	N/A (B.02.08)
RNA Concentration:	160 ng/µl	Result Flagging Color:	
rRNA Ratio [28s / 18s]:	0.2	Result Flagging Label:	RIN N/A

Fragment table for sample 6 : G-1

Name	Start Size [nt]	End Size [nt]	Area	% of total Area
18S	1,621	2,022	54.4	24.6
28S	3,227	3,895	9.5	4.3

 © Copyright 2003 - 2009 Agilent Technologies, Inc.

Assay Class: Eukaryote Total RNA Nano
Data Path: C:\...Eukaryote Total RNA Nano_DE13804083_2014-04-15_13-05-22.xad

Created: 15/04/2014 1:05:21 PM
Modified: 15/04/2014 1:29:08 PM

Electropherogram Summary Continued ...

J-1

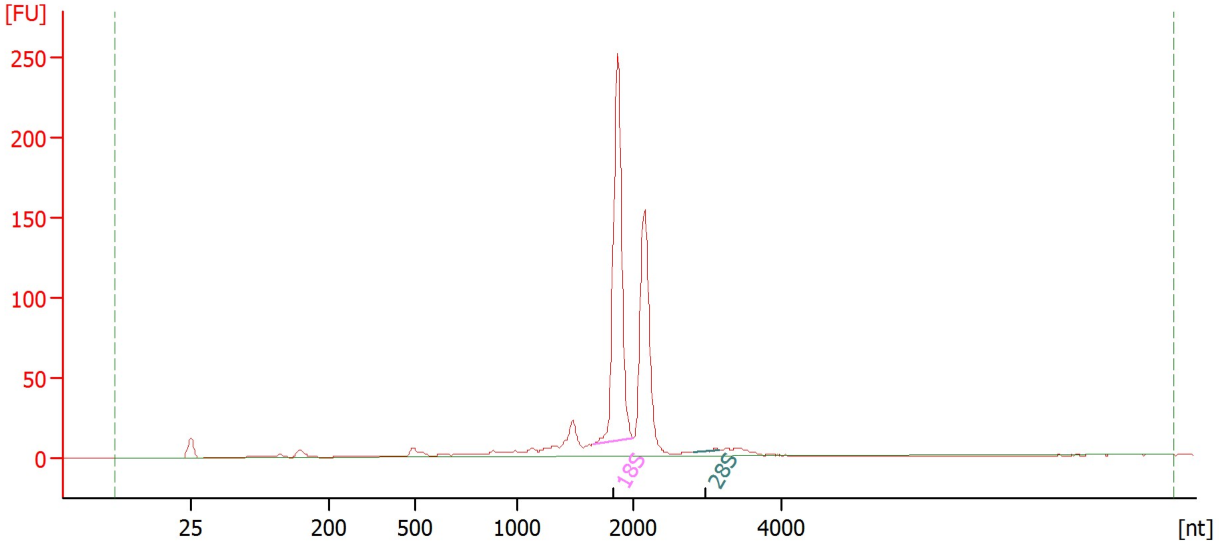

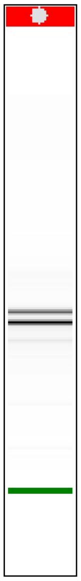

Overall Results for sample 7 : J-1

RNA Area:	630.3	RNA Integrity Number (RIN):	N/A (B.02.08)
RNA Concentration:	456 ng/µl	Result Flagging Color:	
rRNA Ratio [28s / 18s]:	0.0	Result Flagging Label:	RIN N/A

Fragment table for sample 7 : J-1

Name	Start Size [nt]	End Size [nt]	Area	% of total Area
18S	1,645	1,994	254.1	40.3
28S	2,792	3,171	1.3	0.2

 © Copyright 2003 - 2009 Agilent Technologies, Inc.

Assay Class:	Eukaryote Total RNA Nano	Created:	15/04/2014 1:05:21 PM
Data Path:	C:\...Eukaryote Total RNA Nano_DE13804083_2014-04-15_13-05-22.xad	Modified:	15/04/2014 1:29:08 PM

Electropherogram Summary Continued ...

J-1 1

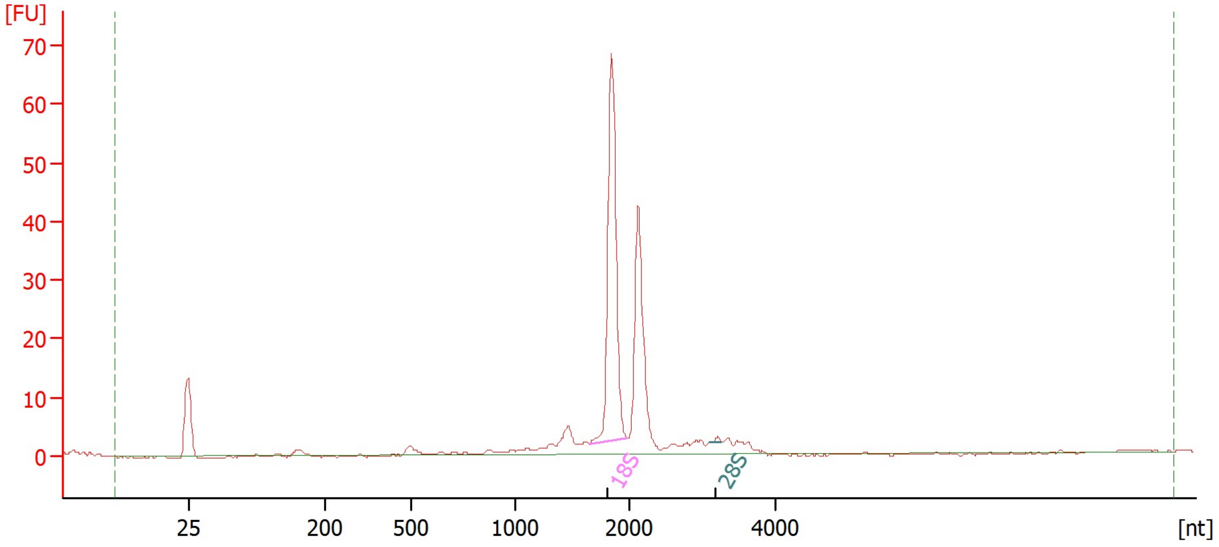

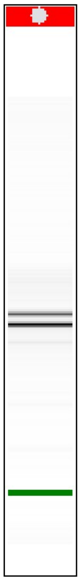

Overall Results for sample 8 : J-1 1

RNA Area:	165.4	RNA Integrity Number (RIN):	N/A (B.02.08)
RNA Concentration:	120 ng/µl	Result Flagging Color:	
rRNA Ratio [28s / 18s]:	0.0	Result Flagging Label:	RIN N/A

Fragment table for sample 8 : J-1 1

Name	Start Size [nt]	End Size [nt]	Area	% of total Area
18S	1,642	1,974	65.8	39.8
28S	3,071	3,262	0.6	0.3

 © Copyright 2003 - 2009 Agilent Technologies, Inc.

Assay Class: Eukaryote Total RNA Nano
Data Path: C:\...Eukaryote Total RNA Nano_DE13804083_2014-04-15_13-05-22.xad

Created: 15/04/2014 1:05:21 PM
Modified: 15/04/2014 1:29:08 PM

Electropherogram Summary Continued ...

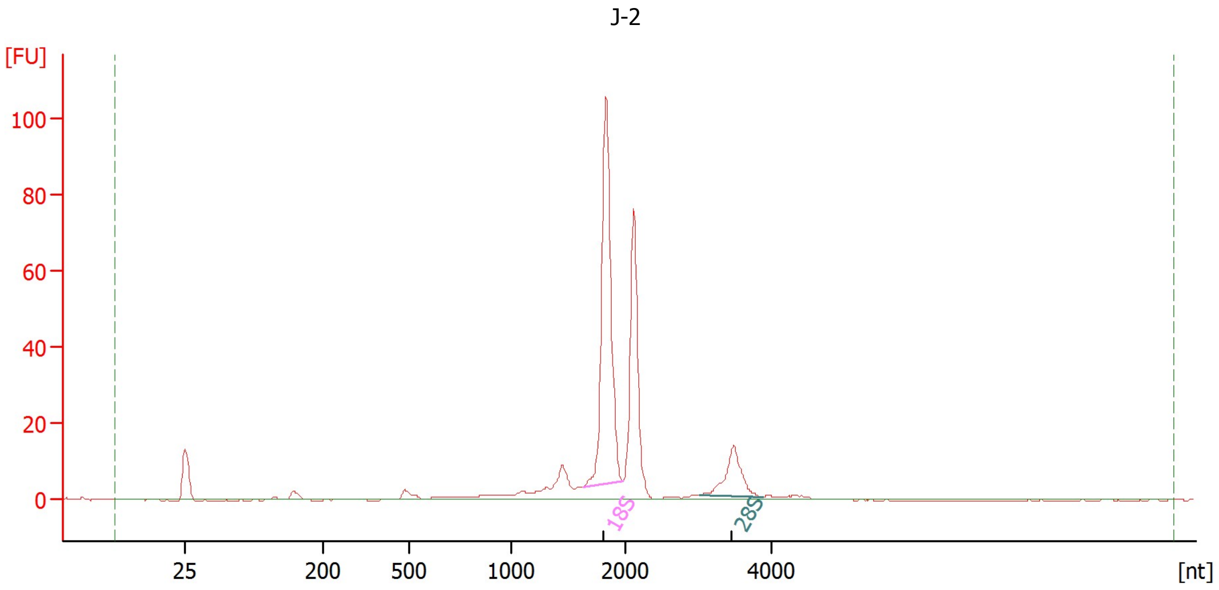

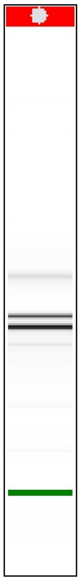

Overall Results for sample 9 : J-2

RNA Area:	290.2	RNA Integrity Number (RIN):	N/A (B.02.08)
RNA Concentration:	210 ng/µl	Result Flagging Color:	
rRNA Ratio [28s / 18s]:	0.2	Result Flagging Label:	RIN N/A

Fragment table for sample 9 : J-2

Name	Start Size [nt]	End Size [nt]	Area	% of total Area
18S	1,620	1,972	122.6	42.2
28S	3,000	3,897	23.0	7.9

© Copyright 2003 - 2009 Agilent Technologies, Inc.

Assay Class:	Eukaryote Total RNA Nano	Created:	15/04/2014 1:05:21 PM
Data Path:	C:\...Eukaryote Total RNA Nano_DE13804083_2014-04-15_13-05-22.xad	Modified:	15/04/2014 1:29:08 PM

Electropherogram Summary Continued ...

J-2 1

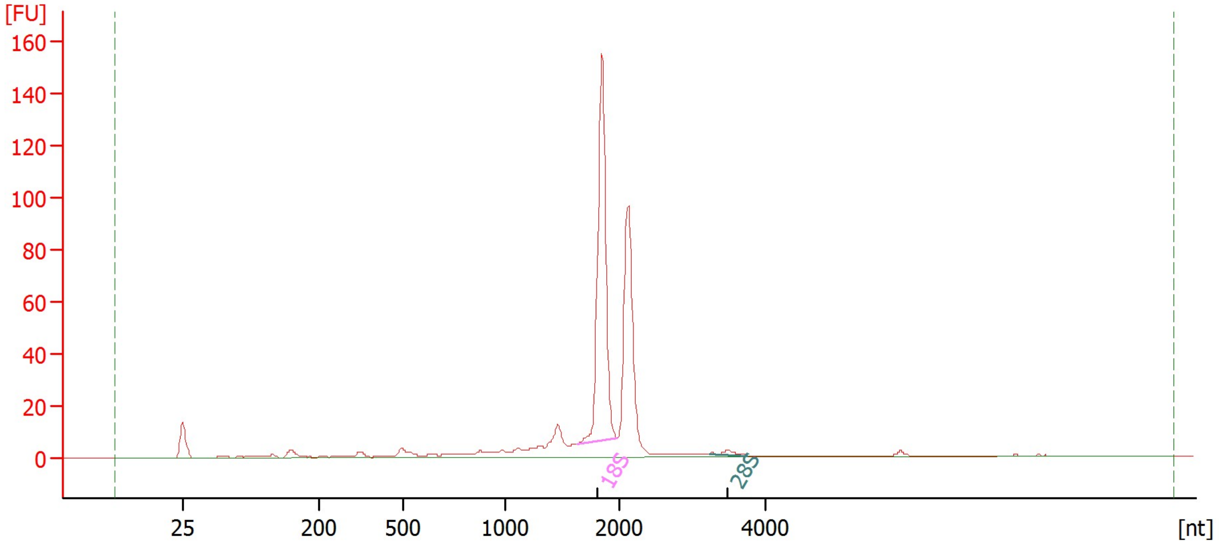

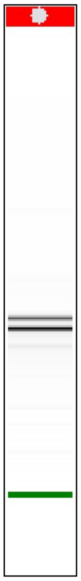

Overall Results for sample 10 : J-2 1

RNA Area:	398.2	RNA Integrity Number (RIN):	N/A (B.02.08)
RNA Concentration:	288 ng/µl	Result Flagging Color:	
rRNA Ratio [28s / 18s]:	0.0	Result Flagging Label:	RIN N/A

Fragment table for sample 10 : J-2 1

Name	Start Size [nt]	End Size [nt]	Area	% of total Area
18S	1,633	1,969	154.3	38.7
28S	3,224	3,772	3.3	0.8

© Copyright 2003 - 2009 Agilent Technologies, Inc.

Assay Class: Eukaryote Total RNA Nano
Data Path: C:\...Eukaryote Total RNA Nano_DE13804083_2014-04-15_13-05-22.xad

Created: 15/04/2014 1:05:21 PM
Modified: 15/04/2014 1:29:08 PM

Electropherogram Summary Continued ...

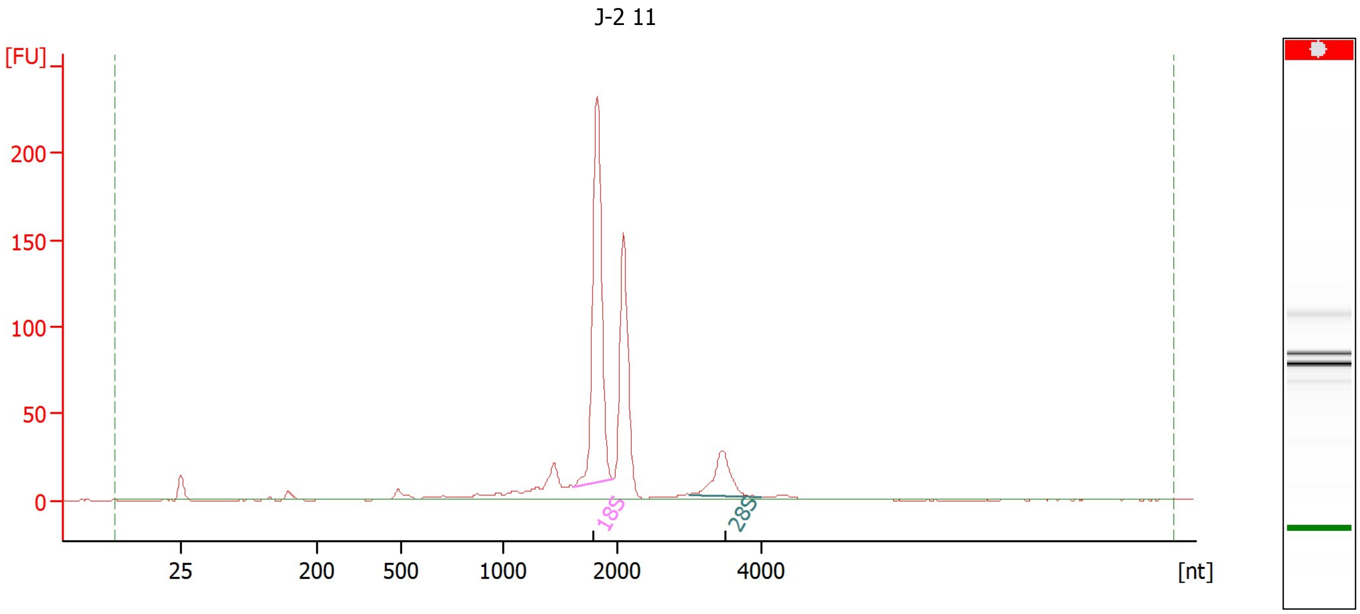

Overall Results for sample 11 : J-2 11

RNA Area:	651.7	RNA Integrity Number (RIN):	N/A (B.02.08)
RNA Concentration:	472 ng/µl	Result Flagging Color:	
rRNA Ratio [28s / 18s]:	0.2	Result Flagging Label:	RIN N/A

Fragment table for sample 11 : J-2 11

Name	Start Size [nt]	End Size [nt]	Area	% of total Area
18S	1,613	1,959	250.5	38.4
28S	3,003	3,997	47.3	7.3

 © Copyright 2003 - 2009 Agilent Technologies, Inc.

Assay Class: Eukaryote Total RNA Nano
Data Path: C:\...Eukaryote Total RNA Nano_DE13804083_2014-04-15_13-05-22.xad

Created: 15/04/2014 1:05:21 PM
Modified: 15/04/2014 1:29:08 PM

Electropherogram Summary Continued ...

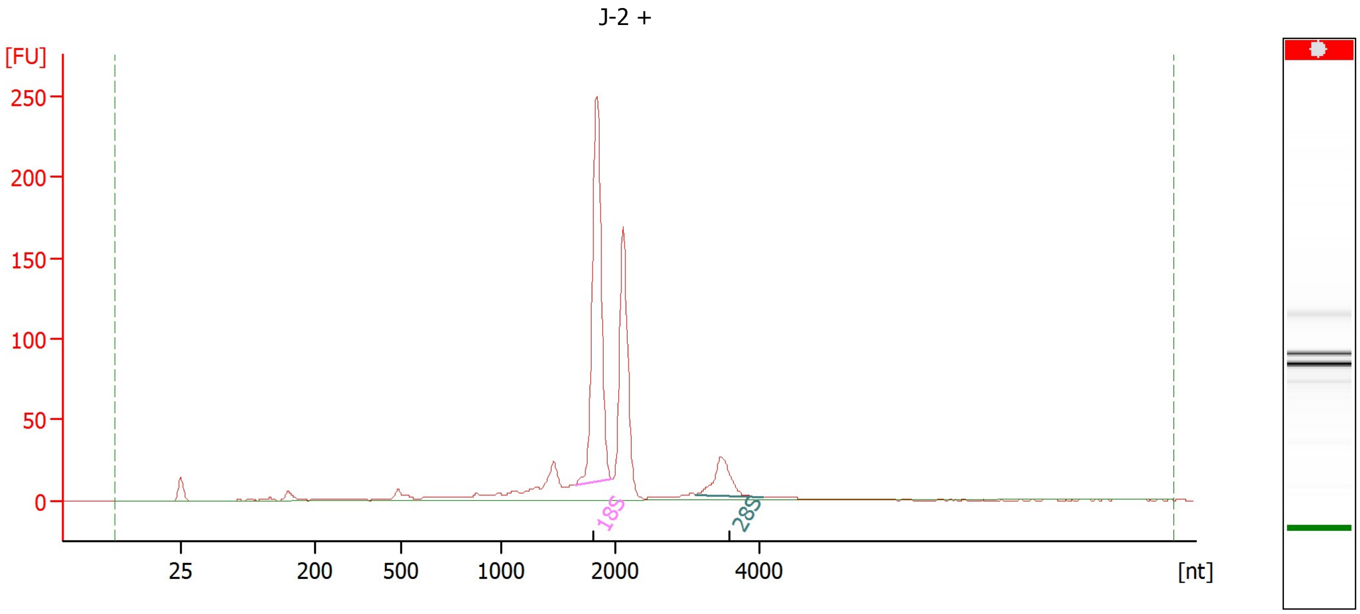

Overall Results for sample 12 : J-2 +

RNA Area:	738.0	RNA Integrity Number (RIN):	N/A (B.02.08)
RNA Concentration:	534 ng/µl	Result Flagging Color:	
rRNA Ratio [28s / 18s]:	0.2	Result Flagging Label:	RIN N/A

Fragment table for sample 12 : J-2 +

Name	Start Size [nt]	End Size [nt]	Area	% of total Area
18S	1,639	1,967	271.0	36.7
28S	3,107	4,044	43.7	5.9

© Copyright 2003 - 2009 Agilent Technologies, Inc.

Assay Class: Eukaryote Total RNA Nano
Data Path: C:\...Eukaryote Total RNA Nano_DE13804083_2014-04-15_13-05-22.xad
Created: 15/04/2014 1:05:21 PM
Modified: 15/04/2014 1:29:08 PM

Gel Image

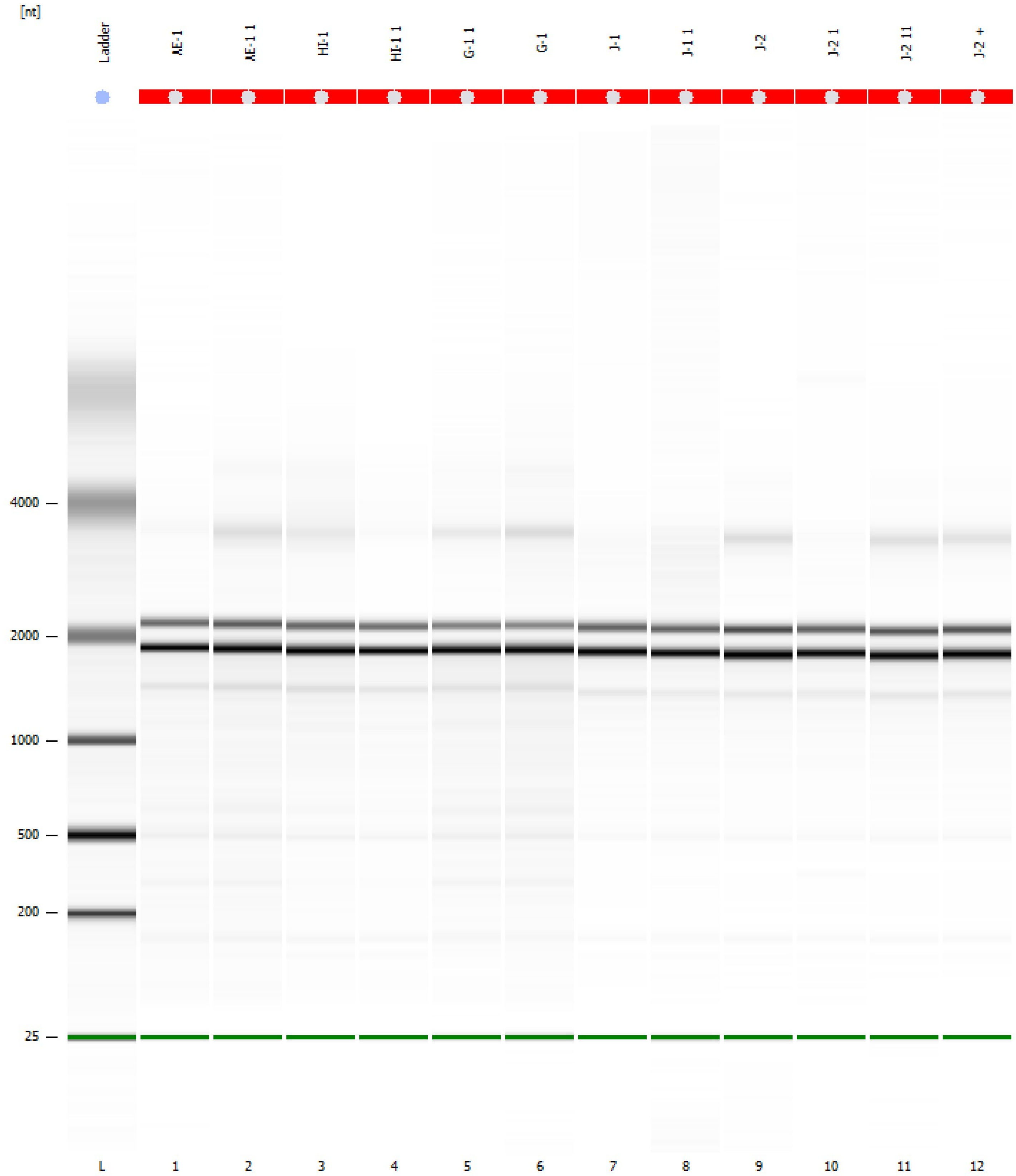

 © Copyright 2003 - 2009 Agilent Technologies, Inc.
